# Comparative efficacy of neuroprotective agents for improving neurological function and prognosis in acute ischemic stroke: a network meta-analysis

**DOI:** 10.3389/fnins.2024.1530987

**Published:** 2025-01-06

**Authors:** Yuchen Wang, Mengqi Li, Yuye Jiang, Qiuhong Ji

**Affiliations:** ^1^Department of Neurology, Affiliated Hospital of Nantong University, Nantong, China; ^2^Medical School of Nantong University, Nantong, China

**Keywords:** neuroprotective agents, network meta-analysis, N-butylphthalide, Edaravone, neurological function, stroke rehabilitation

## Abstract

**Background:**

Ischemic stroke is the second leading cause of death and the third leading cause of combined disability and mortality globally. While reperfusion therapies play a critical role in the management of acute ischemic stroke (AIS), their applicability is limited, leaving many patients with significant neurological deficits and poor prognoses. Neuroprotective agents have garnered attention for their potential as adjunct therapies; however, their relative efficacy remains unclear. This study utilized a network meta-analysis (NMA) to systematically compare the efficacy of neuroprotective agents in improving neurological function and prognosis in stroke patients.

**Methods:**

This study adhered to PRISMA guidelines and the Cochrane Handbook for systematic reviews. Randomized controlled trials (RCTs) were identified through comprehensive searches of the PubMed, Embase, and Cochrane Library databases. Two independent reviewers conducted the selection process, data extraction, and quality assessment. Outcomes included 90-day modified Rankin Scale (90d-mRS), change of National Institutes of Health Stroke Scale score from baseline to 90-day/14-day/7-day (90d/14d/7d-NIHSS) and 90-day/14-day Barthel Index (90d/14d-BI). Data analyses were performed using RevMan 5.4 and Stata 14.0.

**Results:**

A total of 42 RCTs involving 12,210 participants were included in this analysis. The interventions assessed included Cerebrolysin, Citicoline, Edaravone, Edaravone Dextranol, Human urinary kallidinogenase, Minocycline, Nerinetide, Butylphthalide, Vinpocetine, and Control. The NMA results demonstrated that NBP ranked highest for the 90d-mRS, 90d-NIHSS, 14d-NIHSS, and 14d-BI outcomes. Edaravone was found to be the most effective intervention for the 7d-NIHSS and 90d-BI outcomes.

**Conclusion:**

The findings of this study indicate that different neuroprotective agents exhibit distinct advantages at specific stages of recovery. NBP showed outstanding performance in improving 90d-mRS and 90d-NIHSS, underscoring its potential in long-term rehabilitation. Edaravone demonstrated significant superiority in 7d-NIHSS scores, highlighting its role in early neuroprotection. These results provide valuable insights for individualized clinical treatment. To further validate the efficacy and safety of neuroprotective agents, future studies should involve larger sample sizes and conduct multicenter, large-scale randomized controlled trials.

**Systematic review registration:**

https://www.crd.york.ac.uk/prospero/display_record.php?RecordID=601346, identifier CRD42024601346.

## 1 Introduction

The 2019 Global Burden of Disease (GBD) study identified stroke as the second leading cause of death and the third leading cause of combined disability and mortality globally (GBD, 2021). Without immediate intervention, the projected global mortality from stroke is expected to rise by 50% by 2050, resulting in approximately 9.7 million deaths annually, and economic losses may reach as high as $2.3 trillion (Feigin et al., 2022). Each year, over 7.6 million new cases of ischemic stroke are reported, with more than 77 million individuals affected globally (GBD, 2021). Acute ischemic stroke (AIS) is an acute medical emergency characterized by a sudden onset of focal neurological deficits due to impaired cerebral blood flow, typically caused by a range of cerebrovascular factors (Walter, 2022). This ischemic disruption triggers a cascade of pathological events, ultimately resulting in neuronal injury. Current treatment options for AIS are primarily limited to reperfusion therapies, such as intravenous thrombolysis and endovascular thrombectomy (Goyal et al., 2016; Menon et al., 2022). However, strict eligibility criteria and time constraints limit these therapies to only a subset of patients. To address this gap, several therapeutic strategies have been developed to target the ischemic pathophysiological cascade, aiming to prevent irreversible tissue damage. Among these, neuroprotective agents have garnered significant attention (Yanık and Yanık, 2024). Clinically, these agents are frequently employed as adjuncts to standard AIS treatments, and additional neurorestorative approaches are being explored. Numerous neuroprotective drugs have demonstrated promising outcomes in preclinical studies; however, despite initial evidence, the comparative efficacy of these agents or their combinations in AIS remains inconclusive.

Several neuroprotective agents, including Citicoline (Agarwal and Patel, 2017; Secades et al., 2016; Shi et al., 2016), Cerebrolysin (Bornstein et al., 2018; Wan et al., 2024), Minocycline (Malhotra et al., 2018), and Vinpocetine (Panda et al., 2022), have demonstrated efficacy in enhancing neurological function and prognosis in stroke patients, as indicated by previous meta-analyses. However, traditional meta-analyses are limited by their focus on pairwise comparisons, thereby restricting the breadth of interventions assessed. In contrast, network meta-analysis (NMA) enables a comprehensive evaluation and ranking of multiple interventions concurrently (Rouse et al., 2017). This study aims to utilize NMA to compare the efficacy of various neuroprotective agents in improving neurological function and prognosis for ischemic stroke patients. Despite the advancements in reperfusion therapies, many stroke patients continue to experience significant neurological impairments. Neuroprotective agents serve as potential adjuncts to these treatments, yet their relative effectiveness remains ambiguous. Accordingly, we conducted a systematic analysis of randomized controlled trial (RCT) data to evaluate the effects of various neuroprotective interventions, including Nerinetide, Human Urinary Kallidinogenase (HUK), Edaravone, Edaravone Dextranol, Vinpocetine, Butylphthalide, Minocycline, Citicoline, and Cerebrolysin, on key early and long-term outcomes such as the modified Rankin Scale (mRS), National Institutes of Health Stroke Scale (NIHSS), and Barthel Index (BI) scores. This study aims to elucidate the relative efficacy of these neuroprotective agents in enhancing neurological function and prognosis at different recovery phases in ischemic stroke patients.

## 2 Materials and methods

This study adheres to the Preferred Reporting Items for Systematic Reviews and Meta-Analyses (PRISMA) guidelines (Page et al., 2021) and follows the Cochrane Handbook for Systematic Reviews of Interventions to ensure methodological rigor. Furthermore, the network meta-analysis was pre-registered in PROSPERO under the registration number CRD42024601346.

### 2.1 Search strategy

Two authors independently conducted a comprehensive search of PubMed, Embase, and the Cochrane Library to identify randomized controlled trials (RCTs) and crossover RCTs assessing the efficacy of neuroprotective agents in enhancing neurological function and prognosis in ischemic stroke patients. The search period covered all records from the inception of these databases until September 31, 2024. A combination of Boolean operators, Medical Subject Headings (MeSH), and free-text terms was utilized, incorporating search terms such as “Stroke,” “Cerebrovascular accident,” “Brain Vascular Accident,” “hemiplegia,” “apoplexy,” “CVA,” as well as specific neuroprotective agents including “Nerinetide,” “human urinary kallidinogenase,” “Edaravone,” “Vinpocetine,” “Butylphthalide,” “Minocycline,” “Citicoline,” and “Cerebrolysin.” Detailed search strategies for PubMed are available in Supplementary Table 1.

### 2.2 Inclusion criteria

The inclusion criteria were established in accordance with the PICOS framework (Population, Interventions, Comparators, Outcomes, and Study Design) (Hutton et al., 2015).

#### 2.2.1 Inclusion criteria

1. Population: Adults (≥18 years) diagnosed with acute ischemic stroke, confirmed through neuroimaging (including CT, MRI, etc.).
2. Interventions: Nerinetide (NA-1), Hman urinary kallidinogenase (HUK), Edaravone, Edaravone Dextranol, Vinpocetine, N-butylphthalide (NBP), Minocycline, Citicoline and Cerebrolysin.
3. Comparators: The control group included placebo or other neuroprotective agents. Both the intervention group and the control group received standard treatment. Standard care is defined as including both non-neuroprotective pharmacological and non-pharmacological treatments. These encompass interventions aimed at improving cerebral blood flow, such as intravenous thrombolysis, endovascular therapy, antiplatelet therapy, anticoagulant therapy, and fibrinolytic therapy, as well as symptomatic treatments. Symptomatic treatments include respiratory support and oxygen supplementation, cardiac monitoring, temperature regulation, blood pressure management, blood glucose control, and lipid level management, among others.
4. Outcomes: The primary outcome indicator was 90-day modified Rankin Scale score (90d-mRS). The secondary outcome indicators included change of National Institutes of Health Stroke Scale score from baseline to 90-day, 14-day, 7-day (90d/14d/7d-NIHSS) and 90-day, 14-day Barthel Index score (90d/14d-BI).

Detailed scoring criteria for mRS, NIHSS and BI are available in Supplementary Tables 2–4.

5. Study designs: RCTs or crossover RCTs.

#### 2.2.2 Exclusion criteria

1. Patients with hemorrhagic stroke or transient ischemic attack (TIA). Pediatric patients or individuals with pre-existing severe neurological deficits.
2. Conference abstracts, study protocols, reviews, meta-analyses, dissertations, and non-randomized controlled trials (e.g., case reports, observational studies, cross-sectional studies, and studies without control groups).
3. Studies lacking any of the primary or secondary outcome measures.
4. Studies where more than 20% of patients discontinued treatment midway.
5. Studies that could not be downloaded.
6. Studies with incomplete outcome data and no response from the authors after three attempts to contact them.
7. Duplicate publications.

### 2.3 Study selection

First, two authors (Y.C.W and M.Q.L) used Endnote X9 software to remove duplicate articles. Then, they screened titles and abstracts to exclude articles that did not meet the inclusion criteria. Finally, they reviewed the full texts to select articles that met the eligibility criteria. In case of disagreements during the review process, the two authors resolved them through discussion or consultation with a third author (Y.Y.J).

### 2.4 Data extraction

Two authors independently reviewed all articles and extracted the relevant data. The extracted data encompassed basic publication details, including the first author’s name, year of publication, country of study, participant characteristics (age and sample size), intervention details (type, administration method, dosage, and treatment duration), as well as baseline and final outcome measures (mRS, NIHSS, and BI) for calculating change scores. The collected data were entered into an Excel spreadsheet and subsequently cross-checked by both authors. In cases where discrepancies occurred during data extraction, a third author was consulted to resolve the differences through discussion.

### 2.5 Quality assessment

Two authors independently evaluated the risk of bias in the included studies using the Cochrane Risk of Bias Tool (Savović et al., 2014). This tool assesses seven key domains: (1) Random sequence generation, (2) Allocation concealment, (3) Blinding of participants and personnel, (4) Blinding of outcome assessment, (5) Incomplete outcome data, (6) Selective reporting, and (7) Other sources of bias. Each domain was classified as having “low risk,” “unclear risk,” or “high risk” based on the available evidence. In instances where disagreements occurred during the assessment, a third author was consulted to facilitate consensus.

### 2.6 Statistical analysis

Odds ratios (ORs) for binary variables and mean differences (MD) for continuous variables were employed as effect measures, with 95% confidence intervals (CI) provided for each estimate. In cases where different methods or scales were utilized to measure the same outcome, standardized mean differences (SMD) were calculated instead of MD. The differences and standard deviations (SD) for continuous outcome variables before and after treatment were computed according to the guidelines outlined in Section 16.1.3.2 of the Cochrane Handbook version 5.0.2. Statistical analyses were conducted using Stata version 14.0 to perform the network meta-analysis (NMA) and generate various visual representations, including network diagrams for eligible comparisons, Surface Under the Cumulative Ranking (SUCRA) curves, and funnel plots for assessing publication bias (Shim et al., 2017). When loops of evidence existed among interventions, global inconsistency was first evaluated. If *p* > 0.05, the inconsistency was deemed non-significant, and the consistency model was selected. Local inconsistency was assessed using the node-splitting method. SUCRA values were used to rank the interventions, with higher SUCRA values (closer to 100%) indicating better efficacy.

Subgroup analyses were conducted using RevMan 5.4 software. The 95% confidence interval (CI) was employed to represent the statistical effect size. If no significant heterogeneity was detected between studies (*p* ≥ 0.1, I^2^ ≤ 50%), a fixed-effects model was applied. Otherwise, a random-effects model was utilized (*p* < 0.1, I^2^ > 50%) (Tufanaru et al., 2015). To assess the robustness of the results, subgroup analyses were performed to identify potential sources of heterogeneity. Funnel plots with adjustments were employed to evaluate publication bias for each outcome measure in the included studies.

## 3 Results

### 3.1 Search results

**FIGURE 1:**
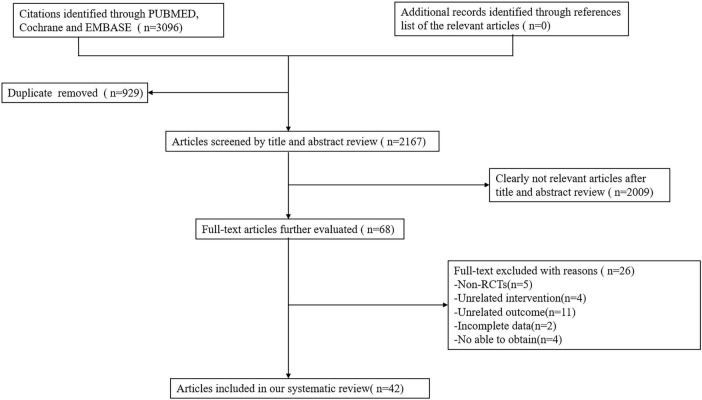
Flow diagram of the eligible studies selection process.

A systematic search of three databases was conducted based on predefined inclusion and exclusion criteria, initially identifying 3,096 articles. After removing duplicates, 2,167 articles remained. Titles and abstracts were then screened, resulting in the exclusion of articles that did not meet the inclusion criteria, reducing the number to 68 articles. Following a full-text review, an additional 26 articles were excluded. Ultimately, 42 randomized controlled trials (RCTs) were included in the final analysis. Figure 1 depicts the screening and selection process of the articles.

### 3.2 Characteristics of the included studies

A total of 42 studies published between 2001 and 2023, involving 12,210 patients, were included in this analysis. All studies were randomized controlled trials (RCTs). In terms of geographical distribution, 47.6% of the included studies originated from China, 14.2% from India, 4.8% each from Russia and Iran, and 2.4% each from the United States, Germany, Canada, New Zealand, Australia, Japan, Israel, Iraq, Serbia, Croatia, Romania, and Austria. The majority of studies were conducted in China (20/42), followed by India (6/42), Russia (2/42), Iran (2/42), and one study each from the other aforementioned countries. The outcome measures assessed included: NBP (11 studies), Cerebrolysin (8 studies), Citicoline (7 studies), Edaravone (6 studies), HUK (5 studies), Minocycline (5 studies), Vinpocetine (4 studies), Edaravone Dextranol (2 studies), and NA-1 (1 study). Detailed characteristics of the included studies are provided in Table 1 (Al Mudhafar et al., 2019; Amiri-Nikpour et al., 2014; Amiri-Nikpour et al., 2015; Belova et al., 2017; Chen L. et al., 2019; Cui et al., 2013; Dávalos et al., 2012; Feigin et al., 2001; Fu et al., 2024; Ghosh et al., 2015; Guo et al., 2021; Hill et al., 2020; Imai et al., 2006; Khasanova and Kalinin, 2023; Kohler et al., 2013; Lampl et al., 2007; Lang et al., 2013; Li et al., 2015; Li et al., 2024; Lou et al., 2024; Mehta et al., 2019; Mitrović et al., 2023; Mittal et al., 2012; Ni et al., 2020; Padma Srivastava et al., 2012; Poljakovic et al., 2021; Sharma et al., 2011; Shinohara et al., 2009; Singh et al., 2021; Song et al., 2018; Stan et al., 2017; Tang and Li, 2019; Wang et al., 2023; Wang et al., 2021; Wang and Che, 2022; Xu et al., 2021; Xue et al., 2016; Yan et al., 2017; Zhang et al., 2017; Zhang et al., 2018; Zhang et al., 2016; Zhou et al., 2022).

**TABLE 1:** Characteristics of included studies.

References	Country	Sample size (E/C)	Intervention protocol (E/C)	Age (E/C, year)	Intervention				Outcomes
					**Duration (day)**	**Dosage**	**Usage**	**Frequency**	
Hill et al., 2020	Canada	549/556	NA-1/CON	71.5 (61.1, 79.7)/ 70.3 (60.4, 80.1)	1	2.6 mg/kg, 270 mg (max)	iv	once	①
Zhang et al., 2018	China	30/30	V/CON	60.3 ± 11.4/ 59.8 ± 13.1	14	30 mg	ivgtt	qd	③
Belova et al., 2017	Russia	100/64	V/CON	58 ± 5/ 57 ± 6	90	10 mg	ivgtt (1–7d)/ po (8–90d)	qd (1–7d)/ tid (8–90d)	①
Zhang et al., 2016	China	469/141	V/CON	63.22 ± 11.76/ 60.02 ± 12.31	7	30 mg	ivgtt	qd	①
Feigin et al., 2001	New Zealand	15/15	V/CON	60.8 (48.71)/ 57.9 (41.79)	90	10 mg/ 30 mg	ivgtt (1–7d)/ po (8–90d)	qd	①
Amiri-Nikpour et al., 2015	Iran	26/27	M/CON	65.23 ± 8.99/ 66.52 ± 7.80	5	200 mg	po	qd	② ③
Kohler et al., 2013	Australian	47/48	M/CON	67.7 (11.0)/ 67.9 (16.3)	0.5	100 mg*5	iv	once	① ④ ⑤
Padma Srivastava et al., 2012	India	23/27	M/CON	52.7 ± 15.3/ 57 ± 14.2	5	200 mg	po	qd	① ② ③ ⑤
Lampl et al., 2007	Israel	74/77	M/CON	67.2 ± 11.1/ 66.2 ± 11.1	5	200 mg	po	qd	① ② ③ ⑤
Li et al., 2024	China	42/38	HUK/CON	56.8 ± 11.1/ 60.8 ± 9.4	14	0.15 PNA	ivgtt	qd	③ ⑤
Guo et al., 2021	China	19/19/19	HUK/NBP/CON	65.95 ± 10.64/ 68.16 ± 12.18/ 69 ± 12.45	14	0.15 PNA HUK/ 25 mg NBP	ivgtt	qd	①
Chen L. et al., 2019	China	58/55	HUK/CON	59 (36.77)/ 60 (39.78)	10	0.15 PNA	ivgtt	qd	① ②
Song et al., 2018	China	21/19	HUK/CON	58.10 ± 13.66/ 60.88 ± 11.85	14	0.15 PNA	ivgtt	qd	④
Li et al., 2015	China	55/55	HUK/CON	63.23 ± 9.09/ 63.13 ± 8.43	14	0.15 PNA	ivgtt	qd	④
Fu et al., 2024	China	450/464	ED/CON	64.1 (56.0, 69.8)/ 64.6 (57.1, 71.4)	14	36 mg	Sublingual	bid	③
Xu et al., 2021	China	585/580	ED/E	62.96 (55.38, 68.96)/ 62.86 (55.72, 70.12)	14	37.5 mg ED/ 30 mg E	ivgtt	q12h	① ③
Sharma et al., 2011	India	25/25	E/CON	58.12 ± 10.79/ 56.0 ± 8.1 5	14	30 mg	ivgtt	bid	① ⑤
Shinohara et al., 2009	Japan	199/202	E/CON	68.48 ± 11.0/ 69.1 ± 10.8	14	30 mg	ivgtt	bid	①
Imai et al., 2006	America	19/19	E/CON	74.9 ± 12.1/ 73.7 ± 13.6	7	30 mg	ivgtt	bid	④
Lou et al., 2024	China	49/49	NBP/CON	62.86 ± 6.62/ 63.67 ± 6.91	14	25 mg	ivgtt	bid	③ ⑥
Wang et al., 2023	China	607/609	NBP/CON	66 (56.72)/ 66 (57.74)	14	25 mg	ivgtt	bid	① ②
Wang and Che, 2022	China	50/50	NBP/CON	63.22 ± 4.06/ 62.13 ± 5.46	14	25 mg	ivgtt	bid	③ ⑥
Zhou et al., 2022	China	164/148	NBP/CON	54 ± 4.15/ 55 ± 2.75	14	25 mg	ivgtt	bid	③ ⑥
Wang et al., 2021	China	60/60	NBP/CON	76.94 ± 8.29/ 75.23 ± 12.15	14	25 mg	ivgtt	bid	③ ⑥
Tang and Li, 2019	China	76/60	NBP/CON	63 ± 6/ 64 ± 6	14	25 mg	ivgtt	bid	③ ⑥
Zhang et al., 2017	China	152/152	NBP/CON	63.89 ± 14.27/ 62.21 ± 12.34	21	0.2 g	po	tid	③ ⑤ ⑥
Yan et al., 2017	China	46/46	NBP/CON	NR/NR	14	0.2 g	po	tid	③
Xue et al., 2016	China	20/20/20	NBP/Ce/CON	67.1 ± 6.3/ 66.5 ± 8.1/ 68.4 ± 4.2	10	25 mg	ivgtt	bid	③ ⑤
Cui et al., 2013	China	159/169	NBP/CON	60.16 ± 10.36/ 9.81 ± 10.08	90	25 mg (1–7d)/ 0.2 g (8–90d)	ivgtt (1–7d)/ po (8–90d)	qd (1–7d)/ tid (8–90d)	④
Singh et al., 2021	India	50/50	Ci/CON	NR/NR	14	500 mg	ivgtt	bid	① ④
Ni et al., 2020	China	466/471	Ci/CON	60.3 (10.31)/62.1 (9.65)	14	320 mg	ivgtt	qd	①
Mehta et al., 2019	India	20/20/20/ 20/20	Ci/Ce/E/ M/CON	59.5/57.3/ 58.8/61.9/64.9	42/14/10/10	500 mg Ci/30 ml Ce/ 30 mg E/200 mg M	ivgtt	bid	① ⑤
Al Mudhafar et al., 2019	Iraq	Ci/CON	35/38	NR/NR	63	1000 mg	po	qd	①
Ghosh et al., 2015	India	Ci/CON	50/50	60.04/64.02	30	1 g (1–5d)/ 0.5 g (6–30d)	ivgtt (1–5d)/ po(6–30d)	q12h(1–5d)/ tid (6–30d)	⑤
Dávalos et al., 2012	Germany	Ci/CON	1148/1150	72.9 ± 11.8/ 72.8 ± 12.1	42	1 g (1–3d)/ 0.5 g (4–42d)	ivgtt (1–3d)/ po (4–42d)	q12h (1–3d)/ tid (4–42d)	① ②
Mittal et al., 2012	India	Ci/CON	24/22/25	54.83/ 57.36/55.6	42	500 mg	po	bid	① ②
Khasanova and Kalinin, 2023	Russia	Ce/CON	117/201	63.5 (56,71)/68 (60,77)	14	30 ml	ivgtt	qd	① ③
Mitrović et al., 2023	Serbia	Ce/CON	30/30	55.7 ± 11.2/ 57.5 ± 11.2	21	30 ml	ivgtt	qd	② ⑤
Poljakovic et al., 2021	Croatia	Ce/CON	23/21	76.0 ± 9.8/ 72.7 ± 10.6	14–21	30 ml	ivgtt	qd	① ④
Stan et al., 2017	Romania	Ce/CON	29/30	62.96 ± 10.9/ 65.24 ± 11.1	10	30 ml	ivgtt	qd	① ② ⑤
Amiri-Nikpour et al., 2014	Iran	Ce/CON	22/21	60 ± 9.6/ 60.1 ± 10.0	10	30 ml	ivgtt	qd	②
Lang et al., 2013	Austria	Ce/CON	55/59	65.5 ± 11.30/ 67.0 ± 10.56	10	30 ml	ivgtt	qd	② ⑤

E, experimental group; C, control group; CON, control; Ce, cerebrolysin; Ci, citicoline; E, Edaravone; ED, Edaravone Dextranol; HUK, human urinary kallidinogenase; M, minocycline; NA-1, nerinetide; NBP, N-butylphthalide; V, vinpocetine. ①90-day mRS; ②: Change of NIHSS from baseline to 90-day; ③: Change of NIHSS from baseline to 14-day; ④: Change of NIHSS from baseline to 7-day; ⑤: 90-day BI; ⑥14-day BI.

### 3.3 Quality evaluation

**FIGURE 2:**
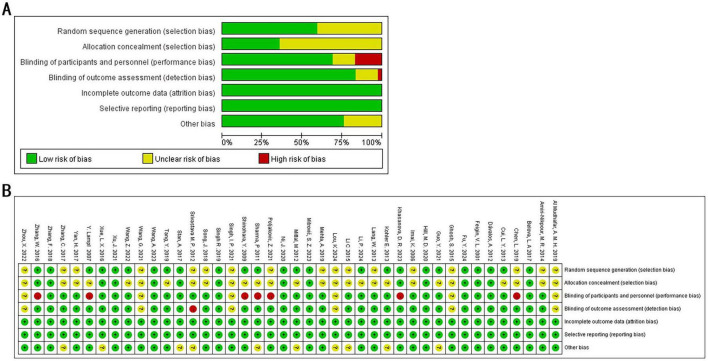
Quality assessment of selected studies by the Cochrane Risk of Bias Tool. **(A)** Risk of bias graph: each risk of bias item presented as percentages across all included studies. **(B)** Risk of bias summary: green indicates a low risk of bias, yellow an unclear risk of bias, and red a high risk of bias.

This study included 42 papers. Although the majority of studies provided detailed descriptions of the randomization process, 17 studies (40.5%) did not specify the methods used for random sequence generation. Fifteen studies (35.7%) were classified as having a low risk of bias concerning allocation concealment. Twenty-nine studies (69.0%) demonstrated a low risk of bias in the blinding of participants and personnel, while 35 studies (83.3%) were assessed as having a low risk of bias for outcome assessment. All 42 studies (100%) were considered to have a low risk of bias concerning incomplete outcome data and selective reporting. With regard to other biases, most studies declared no conflicts of interest; however, 10 studies presented an unclear risk for other potential biases. Detailed results of the bias risk assessment are provided in Figure 2.

### 3.4 Pairwise meta-analysis

Pairwise meta-analyses were conducted to comprehensively compare two interventions at a time. Specifically, six distinct pairwise analyses were performed for the following outcomes: 90-day modified Rankin Scale (mRS), 90-day National Institutes of Health Stroke Scale (NIHSS), 14-day NIHSS, 7-day NIHSS, 90-day Barthel Index (BI), and 14-day BI. A summary of the results is provided in Table 2, while the detailed results of the pairwise meta-analyses are available in Supplementary Figures 1–6.

**TABLE 2:** Pairwise meta-analysis.

Comparison	Number of studies	MD/SMD (95% CI)	Heterogeneity test	
			**I2 (%)**	***p*-value**
**90d-mRS**				
NA-1/Control	1	−0.07 (−0.35, 0.21)	NR	0.62
Vinpocetine/Control	3	−0.96 (−1.33, −0.59)	86	<0.00001
Minocycline/Control	3	−0.88 (−1.15, −0.60)	72	<0.00001
HUK/Control	2	−0.63 (−0.93, −0.33)	0	<0.0001
NBP/Control	2	−0.59 (−0.83, −0.35)	0	<0.00001
Edaravone/Control	2	−0.27 (−0.53, 0.00)	61	0.05
Edaravone Dextranol/Control	1	0.01 (−0.10, 0.12)	NR	0.85
Citicoline/Control	4	−0.33 (−0.47, −0.18)	90	<0.0001
Cerebrolysin/Control	4	−0.24 (−0.48, 0.00)	0	0.05
HUK/NBP	1	−0.10 (−0.46, 0.26)	NR	0.58
Edaravone/Edaravone Dextranol	1	−0.02 (−0.11, 0.07)	NR	0.65
Edaravone/Citicoline	1	0.67 (−0.01, 1.35)	NR	0.05
**90d-NIHSS**				
Minocycline/Control	4	−4.12 (−4.99, −3.25)	40	<0.00001
HUK/Control	1	−1.80 (−2.89, −0.71)	NR	0.001
Edaravone Dextranol/Control	1	0.40 (−0.01, 0.81)	NR	0.06
NBP/Control	2	−1.04 (−1.15, −0.94)	92	<0.00001
Citicoline/Control	3	−1.53 (−2.54, −0.52)	0	0.003
Cerebrolysin/Control	7	−1.91 (−2.40, −1.42)	65	<0.00001
Edaravone/Edaravone Dextranol	1	0.40 (−0.01, 0.81)	NR	0.06
Edaravone/Citicoline	2	0.23 (−2.08, 2.54)	0	0.84
Cerebrolysin/Edaravone	1	−0.66 (−3.54, 2.22)	NR	0.65
Citicoline/Minocycline	1	−2.19 (−4.59, 0.21)	NR	0.07
Edaravone/Minocycline	1	−2.03 (−4.72, 0.66)	NR	0.14
Cerebrolysin/Minocycline	1	−2.19 (−4.59, 0.21)	NR	0.07
**14d-NIHSS**				
Vinpocetine/Control	1	1.50 (−2.52, 5.52)	NR	0.73
HUK/Control	1	−1.00 (−1.58, −0.42)	NR	0.0008
Edaravone Dextranol/Control	1	−0.35 (−0.45, −0.25)	NR	<0.00001
Edaravone/Edaravone Dextranol	1	0.40 (−0.01, 0.81)	NR	0.06
NBP/Control	7	−2.98 (−3.25, −2.70)	98	<0.00001
Cerebrolysin/Control	2	−0.98 (−1.96, 0.00)	94	0.05
Cerebrolysin/NBP	1	0.78 (−1.58, 3.14)	NR	0.52
**7d-NIHSS**				
Minocycline/Control	1	−0.66 (−2.15, 0.83)	NR	0.38
HUK/Control	2	−1.18 (−2.14, −0.23)	0	0.02
Edaravone/Control	1	−4.46 (−6.65, −2.27)	NR	<0.0001
Cerebrolysin/Control	1	−2.44 (−4.58, −0.30)	NR	0.03
**90d-BI**				
Minocycline/Control	3	11.32 (6.92, 15.73)	80	<0.00001
NBP/Control	3	4.85 (2.48, 7.23)	68	<0.0001
Edaravone/Control	2	14.09 (9.19, 18.98)	0	<0.00001
**90d-mRS**				
Citicoline/Control	3	2.04 (0.77, 3.31)	79	0.002
Cerebrolysin/Control	4	9.64 (5.90, 13.38)	60	<0.00001
**90d-BI**				
Cerebrolysin/Citicoline	1	7.67 (1.00, 14.34)	NR	0.02
Cerebrolysin/Edaravone	1	10.55 (4.31, 16.79)	NR	0.0009
Cerebrolysin/Minocycline	1	−1.98 (−8.63, 4.67)	NR	0.56
Citicoline/Edaravone	1	2.88 (−1.62, 7.38)	NR	0.21
Citicoline/Minocycline	1	−9.65 (−14.70, −4.60)	NR	0.0002
Edaravone/Minocycline	1	12.53 (8.06, 17.00)	NR	<0.00001
**14d-BI**				
HUK/Control	1	5.00 (−1.09, 11.09)	NR	0.11
NBP/Control	6	12.95 (12.23, 13.67)	97	<0.00001
Cerebrolysin/Control	1	10.00 (1.11, 18.89)	NR	0.03
Cerebrolysin/NBP	1	0.25 (−8.15, 8.65)	NR	0.95

Red and bold numbers are statistically significant. HUK, human urinary kallidinogenase; NA-1, nerinetide; NBP, N-butylphthalide. NR, not reported.

### 3.5 Network of evidence

**FIGURE 3:**
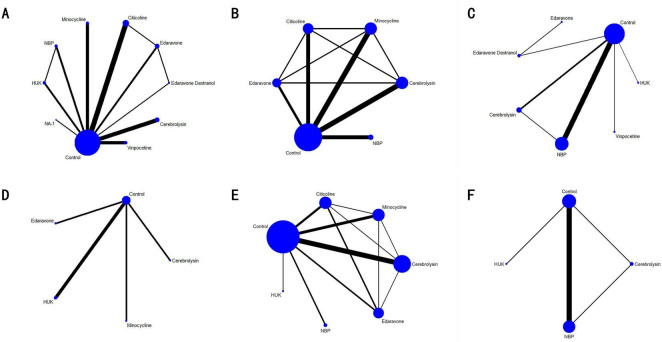
Network meta-analysis diagrams of eligible comparisons. **(A)** 90d-mRS; **(B)** 90d-NIHSS; **(C)** 14d-NIHSS; **(D)** 7d-NIHSS; **(E)** 90d-BI; **(F)** 14d-BI. HUK, human urinary kallidinogenase; NBP, N-butylphthalide.

A total of 42 randomized controlled trials (RCTs) involving 12,210 participants were included to assess the effects of various neuroprotective agents on neurological function and prognosis in ischemic stroke patients. Figure 3 depicts the network meta-analysis diagram for eligible comparisons. Blue solid circles represent the different interventions, and the size of each circle corresponds to the sample size for that intervention. Black lines connecting the blue circles indicate direct comparisons between two interventions, with the thickness of these lines reflecting the number of studies included in the comparisons. This network visualization provides a comprehensive overview of both direct and indirect comparisons among the included interventions.

#### 3.5.1 90-day mRS

**FIGURE 4:**
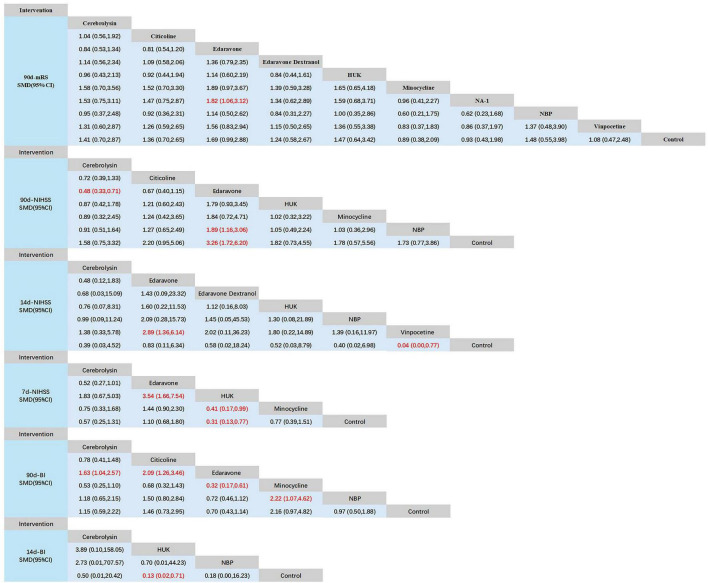
Network meta-analysis of head-to-head comparisons. Red and bold numbers are statistically significant. HUK, human urinary kallidinogenase; NBP, N-butylphthalide.

**FIGURE 5:**
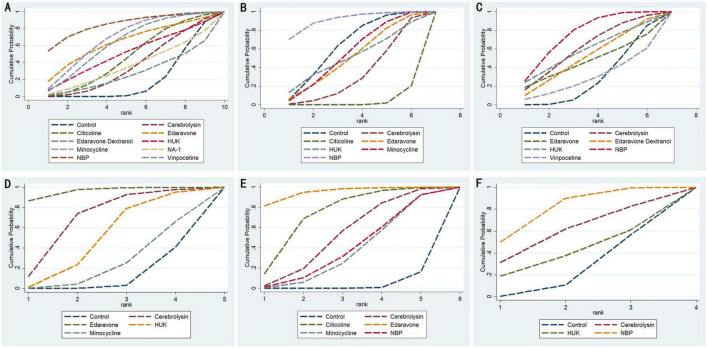
Cumulative probability ranking curve of different interventions. The vertical axis represents cumulative probabilities, while the horizontal axis represents ranks. **(A)** 90d-mRS; **(B)** 90d-NIHSS; **(C)** 14d-NIHSS; **(D)** 7d-NIHSS; **(E)** 90d-BI; **(F)** 14d-BI. HUK, human urinary kallidinogenase; NBP, N-butylphthalide.

A total of 22 studies evaluated the 90-day modified Rankin Scale (mRS), including 10 interventions: Cerebrolysin, Citicoline, Edaravone, Edaravone Dextranol, HUK, Minocycline, NA-1, NBP, Vinpocetine, and a control group, with 10,057 patients included. The inconsistency model showed no significant global inconsistency (*p* = 0.7085, *p* > 0.05; Supplementary Figure 7A), so the consistency model was applied. The node-splitting method indicated good local consistency, as all *p*-values were greater than 0.05 (Supplementary Figure 8). The network meta-analysis (NMA) produced 45 pairwise comparisons. Compared with the control group, Minocycline (SMD = 0.61, 95% CI: 0.22 1.00), HUK (SMD = 0.59, 95% CI: 0.09 1.08), and Vinpocetine (SMD = 0.54, 95% CI: 0.16 0.92) significantly improved 90-day mRS scores for stroke patients. Other pairwise comparisons showed no statistically significant differences (*p* > 0.05; Figure 4). According to SUCRA analysis and ranking, NBP (SUCRA: 85.0%) was likely the most effective intervention for improving 90-day mRS outcomes in stroke patients (Figure 5A and Table 3).

**TABLE 3:** SUCRA values of different interventions for outcomes.

Outcomes	NBP	Edaravone	HUK	Minocycline	Citicoline	ED	Vinpocetine	NA-1	Control
90d-mRS	85.0%^A^	47.0%	50.5%	69.3%	47.0%	27.3%	63.8%	36.3%	19.6%
90d-NIHSS	91.5%^A^	51.2%	51.3%	55.4%	33.1%	NR	NR	NR	3.8%
14d-NIHSS	75.9%^B^	46.5%	85.0%^A^	NR	NR	51.2%	28.9%	NR	27.9%
7d-NIHSS	NR	96.1%^A^	49.9%^B^	NR	NR	NR	NR	NR	11.0%
90d-BI	39.4%	94.9%^A^	NR	36.3%	73.6%^B^	NR	NR	NR	3.4%
14d-BI	79.9%^A^	NR	39.1%^B^	NR	NR	NR	NR	NR	22.4%

A: presents the first-ranking.

B: presents the second-ranking. ED, Edaravone Dextranol; HUK, human urinary kallidinogenase; NA-1, nerinetide; NBP, N-butylphthalide; NR, not reported.

#### 3.5.2 90-day NIHSS

A total of 15 studies evaluated the 90-day NIHSS, involving 7 interventions: Cerebrolysin, Citicoline, Edaravone, HUK, Minocycline, NBP, and placebo. A total of 4,591 patients were included. The inconsistency model showed no significant global inconsistency (*p* = 0.5899, *p* > 0.05; Supplementary Figure 7B), so the consistency model was applied. The node-splitting method indicated good local consistency, as all *p*-values were greater than 0.05 (Supplementary Figure 9). The NMA produced 21 pairwise comparisons. Compared with the control group, Cerebrolysin (SMD = −0.72, 95% CI: −1.10 −0.35), Minocycline (SMD = 0.63, 95% CI: 0.15 1.12), and NBP (SMD = 1.18, 95% CI: 0.54 1.82) significantly improved 90-day NIHSS scores for stroke patients. Other pairwise comparisons showed no statistically significant differences (*p* > 0.05; Figure 4). According to SUCRA analysis and ranking, NBP (SUCRA: 91.5%) was likely the most effective intervention for improving 90-day NIHSS outcomes in stroke patients (Figure 5B and Table 3).

#### 3.5.3 14-day NIHSS

A total of studies evaluated the 14-day NIHSS, involving 7 interventions: Cerebrolysin, Edaravone, Edaravone Dextranol, HUK, NBP, Vinpocetine, and placebo. A total of 4,591 patients were included. The inconsistency model showed no significant global inconsistency (*p* = 0.6412, *p* > 0.05; Supplementary Figure 7C), so the consistency model was applied. The node-splitting method indicated good local consistency, as all *p*-values were greater than 0.05 (Supplementary Figure 10). The NMA produced 21 pairwise comparisons. Compared with the control group, only NBP (SMD = 1.06, 95% CI: 0.27 1.86) significantly improved 14-day NIHSS scores for stroke patients. Other pairwise comparisons showed no statistically significant differences (*p* > 0.05; Figure 4). According to SUCRA analysis and ranking, NBP (SUCRA: 75.9%) was likely the most effective intervention for improving 14-day NIHSS outcomes in stroke patients (Figure 5C and Table 3).

#### 3.5.4 7-day NIHSS

A total of studies evaluated the 7-day NIHSS, involving 5 interventions: Cerebrolysin, Edaravone, HUK, Minocycline, and placebo. The total number of patients included was unspecified. There were no loops in the network, so consistency checks were not required. The NMA produced 10 pairwise comparisons. Compared with placebo, Edaravone (SMD = 1.01, 95% CI: 0.64–1.38) significantly improved 7-day NIHSS scores in stroke patients. Additionally, Edaravone showed significantly greater improvement in 7-day NIHSS compared to HUK (SMD = −0.90, 95% CI: −1.75 −0.05) and Minocycline (SMD = −1.09, 95% CI: −1.99 −0.19). No statistically significant differences were found among the other interventions (*p* > 0.05; Figure 4). According to SUCRA analysis and ranking, Edaravone (SUCRA: 96.1%) was likely the most effective intervention for improving 7-day NIHSS outcomes in stroke patients (Figure 5D and Table 3).

#### 3.5.5 90-day BI

A total of 15 studies evaluated the 90-day Barthel Index (BI), involving 6 interventions: Cerebrolysin, Citicoline, Edaravone, Minocycline, NBP, and placebo, with 4,591 patients included. The inconsistency model showed no significant global inconsistency (*p* = 0.4253, *p* > 0.05; Supplementary Figure 7D), so the consistency model was applied. The node-splitting method indicated good local consistency, as all *p*-values were greater than 0.05 (Supplementary Figure 11). The NMA produced 15 pairwise comparisons. Compared with placebo, Cerebrolysin (SMD = 0.49, 95% CI: 0.06 0.92), Citicoline (SMD = 0.73, 95% CI: 0.26–1.21), and Edaravone (SMD = −1.12, 95% CI: −1.73 −0.52) significantly improved 90-day BI scores in stroke patients. Additionally, Edaravone showed significantly greater improvement in 90-day BI compared to Minocycline (SMD = 0.79, 95% CI: 0.10 1.49) and NBP (SMD = 0.77, 95% CI: 0.01 1.53). No statistically significant differences were found among other pairwise comparisons (*p* > 0.05; Figure 4). According to SUCRA analysis and ranking, Edaravone (SUCRA: 94.9%) was likely the most effective intervention for improving 90-day BI outcomes in stroke patients (Figures 5B, E, and Table 3).

#### 3.5.6 14-day BI

A total of 7 studies evaluated the 14-day Barthel Index (BI), involving 4 interventions: Cerebrolysin, HUK, NBP, and placebo, with 1,073 patients included. The inconsistency model showed no significant global inconsistency (*p* = 0.5182, *p* > 0.05; Supplementary Figure 7E), so the consistency model was applied. The node-splitting method indicated good local consistency, as all *p*-values were greater than 0.05 (Supplementary Figure 12). The NMA produced 6 pairwise comparisons. Compared with placebo, NBP (SMD = −2.00, 95% CI: −3.00 −0.99) significantly improved 14-day BI scores in stroke patients. No statistically significant differences were found among other pairwise comparisons (*p* > 0.05; Figure 4). According to SUCRA analysis and ranking, NBP (SUCRA: 79.9%) was likely the most effective intervention for improving 14-day BI outcomes in stroke patients (Figure 5F and Table 3).

### 3.6 Publication bias

**FIGURE 6:**
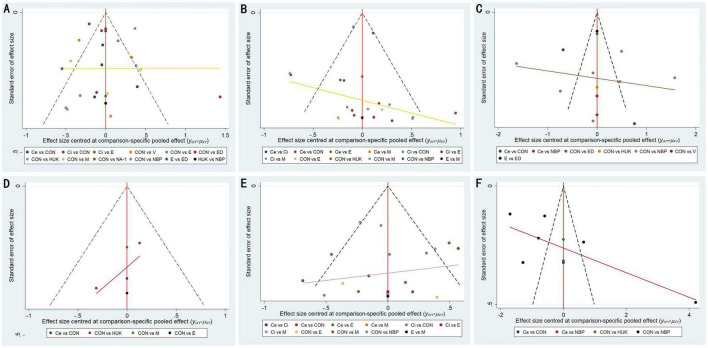
Funnel plot of publication bias. **(A)** 90d-mRS; **(B)** 90d-NIHSS; **(C)** 14d-NIHSS; **(D)** 7d-NIHSS; **(E)** 90d-BI; **(F)** 14d-BI. CON, control; Ce, cerebrolysin; Ci, citicoline; E, Edaravone; ED, Edaravone Dextranol; HUK, human urinary kallidinogenase; M, minocycline; NA-1, nerinetide; NBP, N-butylphthalide; V, vinpocetine.

We used STATA 14.0 to create adjusted funnel plots for comparisons of 90-day mRS, 90-day NIHSS, 14-day NIHSS, 7-day NIHSS, 90-day BI, and 14-day BI (Figure 6). The results showed that the studies were generally centered and exhibited good symmetry, indicating a low likelihood of publication bias.

## 4 Discussion

Stroke is a prevalent acute condition worldwide, leading to significant neurological impairments and poor prognoses for patients. Current treatment strategies for ischemic stroke are primarily limited to vascular recanalization therapies, which benefit only a small subset of patients. Concurrently, the pathophysiological cascade of irreversible tissue damage initiated by cerebral ischemia has become increasingly well understood (Chamorro et al., 2016; Lyden, 2021). As a result, a growing number of neuroprotective agents are under development. Despite these efforts, no neuroprotective strategy has yet been translated into a universally accepted clinical approach (Haupt et al., 2023). Although certain neuroprotective methods have demonstrated promise in preclinical settings, their clinical application largely depends on the experience of clinicians and region-specific treatment practices. There is insufficient evidence to definitively identify the most suitable neuroprotective method for AIS patients. This study utilized a network meta-analysis (NMA) to compare the efficacy of various neuroprotective agents, including Nerinetide, HUK, Edaravone, Edaravone Dextranol, Vinpocetine, NBP, Minocycline, Citicoline, and Cerebrolysin. The analysis incorporated data from 42 RCTs involving 3,017 participants and examined the efficacy of 9 neuroprotective agents. The modified Rankin Scale (mRS), a tool for assessing functional recovery following stroke or neurological injury (Bamford et al., 1989), was selected to evaluate outcomes at 90 days. The National Institutes of Health Stroke Scale (NIHSS), a standardized tool for measuring stroke-related neurological deficits (with higher scores indicating more severe damage)(Yoo et al., 2010), was used to assess changes in NIHSS scores from baseline to days 7, 14, and 90 to evaluate both early and long-term neurological improvements. Additionally, the Barthel Index (BI), which measures a person’s ability to perform activities of daily living (ADL) independently (with higher scores reflecting greater functional independence)(Wade and Collin, 1988), was used to evaluate the impact of neuroprotective agents on early and long-term daily life functions, specifically at 14 and 90 days post-stroke.

The findings of this study revealed significant differences in the efficacy of various neuroprotective agents in enhancing neurological function and prognosis for ischemic stroke patients across different recovery stages. Specifically, NBP showed notable improvements in 90-day mRS, 90-day NIHSS, and 7-day BI compared to conventional treatments, suggesting its broad applicability across different stages of recovery. Evidence suggests that the mechanism of NBP involves enhancing cerebral blood flow and exerting antioxidative effects to protect neurons and delay neuronal death (Chen X.-Q. et al., 2019; Huang et al., 2018), emphasizing its potential clinical significance for the long-term rehabilitation of AIS patients. Edaravone demonstrated notable efficacy in improving 90-day BI scores and 7-day NIHSS. Its significant effect on the 7-day NIHSS suggests its potential role in early neuroprotection, which may be attributed to its antioxidative properties, as Edaravone scavenges free radicals, reduces brain cell damage, and promotes early neurological recovery (Cao et al., 2023; Yamashita and Abe, 2024). Minocycline also showed significant improvements in 90-day modified Rankin Scale (mRS) scores, indicating its potential suitability for long-term intervention. The mechanism of action of Minocycline may involve inhibiting neuroinflammation and protecting the vascular endothelium, thereby mitigating neural damage during the chronic phase (Zhao et al., 2023). Notably, Cerebrolysin demonstrated less efficacy than NBP and Edaravone in improving 90-day and 14-day NIHSS scores, potentially due to its mechanism being more focused on enhancing short-term memory and cognitive function rather than motor function recovery in ischemic stroke patients (Mureşanu et al., 2022; Rejdak et al., 2023). Furthermore, Citicoline has been reported in some studies to effectively improve functional outcomes in patients with AIS. However, two studies reported no significant differences compared to standard treatments (Al Mudhafar et al., 2019; Dávalos et al., 2012). This study found that Citicoline’s efficacy in improving 90-day mRS scores was inferior to that of other neuroprotective agents, suggesting that its application in AIS requires further research and validation.

The findings of this study hold significant clinical value for the treatment of AIS patients. Considering the differences in short- and long-term effects of various drugs, it is recommended that clinicians tailor the choice of neuroprotective agents based on the patient’s specific condition and stage of recovery. First, NBP and Edaravone demonstrated excellent performance in long-term follow-up scores, indicating their potential to improve long-term prognosis after stroke. These agents may be particularly suitable for maintenance therapy following early intervention. Second, Edaravone exhibited superior efficacy in NIHSS scores within the first 7 days compared to other drugs, suggesting its potential for providing rapid neuroprotective effects in acute stroke treatment. Therefore, during the acute phase—especially in the early stages following stroke onset—the use of Edaravone may help maximize neuroprotection and reduce early disability rates (Kimura et al., 2012). Additionally, the results of this study suggest that neuroprotective agents are more effective when administered as early interventions in stroke patients. This observation aligns with the physiological characteristics of brain tissue, which are more responsive to treatment and exhibit greater recovery potential during the early stages. Furthermore, preliminary findings from existing studies indicate that the efficacy of neuroprotective strategies often surpasses that of single-drug approaches in achieving better long-term outcomes (Li et al., 2022; Tran et al., 2022). Given the complexity of neurological recovery and the substantial individual variability observed in stroke patients, future clinical applications should incorporate factors such as stroke type, onset timing, lesion location, and the severity of neurological damage. Such an approach would facilitate the selection of the most suitable drug combinations or individualized treatment plans, thereby maximizing the potential for optimal clinical outcomes.

This study provides preliminary evidence regarding the comparative efficacy of neuroprotective agents through NMA, including improvements in 90-day mRS and NIHSS; however, several limitations remain. First, the included studies exhibited imbalanced sample sizes, with many being small-scale trials, potentially compromising the stability and generalizability of the findings. Second, although NMA facilitates the comparison of multiple interventions, it predominantly relies on indirect comparisons across studies rather than direct head-to-head comparisons among all drugs. Third, this study was unable to fully eliminate potential biases present in the included trials, particularly those related to random sequence generation and allocation concealment. The low methodological quality of certain studies—such as a lack of blinding, high dropout rates, and inadequate randomization—may have influenced the final outcomes. Finally, this study did not conduct a detailed analysis of the long-term safety and adverse effects of neuroprotective agents. Although some trials reported mild adverse effects, such as dizziness and headache, systematic evaluations of adverse events were lacking. Future research should incorporate safety data to further validate the risk-benefit profiles of neuroprotective agents, thereby providing more comprehensive guidance for their safe clinical use. To address the aforementioned limitations, future research should prioritize expanding sample sizes and including multicenter, highly heterogeneous populations to enhance the representativeness and generalizability of findings. Designing head-to-head comparative studies is crucial for clarifying the relative efficacy priorities of different drugs. Additionally, further exploration of the effects of various drug combinations is warranted to evaluate their potential synergistic effects in improving neurological function. While some evidence suggests that early combination therapy with neuroprotective agents may yield superior long-term outcomes compared to monotherapy, the potential side effects and higher costs associated with prolonged combination regimens must be carefully considered. The demand for neuroprotective agents may vary across different recovery stages, making stratified treatment strategies tailored to specific phases a promising avenue for future research. For instance, acute-phase drugs such as Edaravone may be more effective in mitigating early damage, whereas medications like NBP might better support long-term functional recovery during rehabilitation. Finally, given the significant impact of neuroprotective agents on the long-term quality of life for stroke patients, future studies should incorporate more comprehensive assessments of quality of life, including factors such as depression, anxiety, and social participation. Such evaluations would provide a more holistic understanding of the effects of neuroprotective agents on stroke recovery and contribute to the development of optimized post-stroke rehabilitation strategies.

## 5 Conclusion

This network meta-analysis offers a comprehensive comparison of the efficacy of various neuroprotective agents in enhancing neurological function and prognosis for patients with ischemic stroke. The evidence from this study indicates that different drugs provide distinct benefits at specific stages of recovery. NBP demonstrated notable efficacy in improving 90d-mRS and 90d-NIHSS, highlighting its potential in long-term rehabilitation. Edaravone exhibited significant superiority in improving 7d-NIHSS scores, suggesting its potential role in early neuroprotection. These findings offer valuable insights for individualized clinical treatment. To further validate the efficacy and safety of different neuroprotective agents, future research should include larger sample sizes, involve multicenter and large-scale randomized controlled trials, and assess the effects of combination therapies. Such efforts will help confirm and strengthen the findings of this study. In conclusion, this research underscores the substantial value of neuroprotective agents in enhancing neurological function and prognosis for patients with ischemic stroke.

## Data Availability

The original contributions presented in this study are included in this article/Supplementary material, further inquiries can be directed to the corresponding author.
