# Supplementary material for: Comparative efficacy of neuroprotective agents for improving neurological function and prognosis in acute ischemic stroke: a network meta-analysis
Source: Front Neurosci. 2025 Jan 6;18:1530987. doi: 10.3389/fnins.2024.1530987 (PMC11743486; doi:10.3389/fnins.2024.1530987)
Supplement: Supplementary file 1 [file Data_Sheet_1.docx]

Supplementary Material

1. **Supplementary Figures**

**
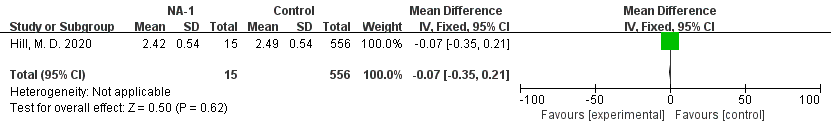
**

**
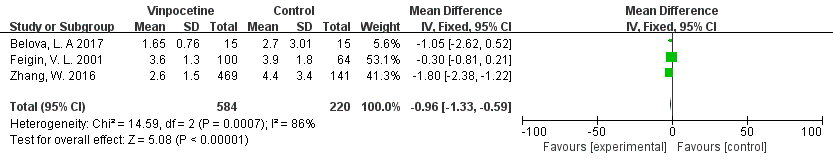

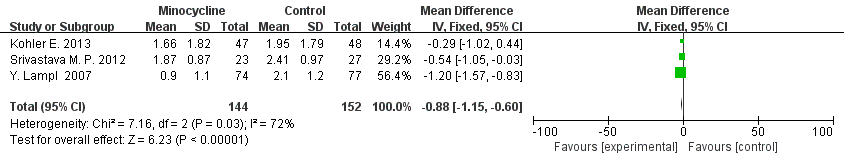

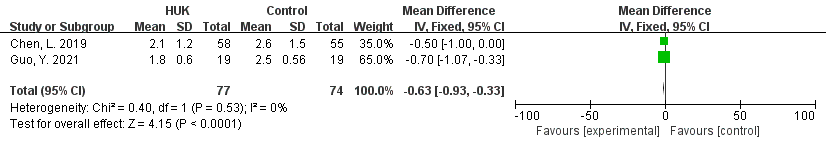

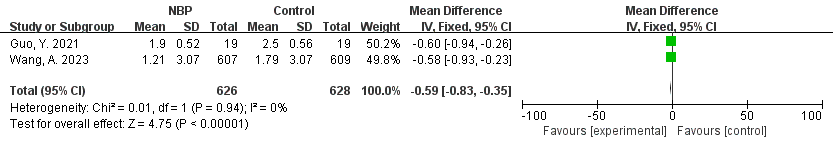

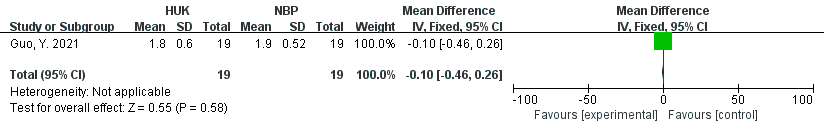

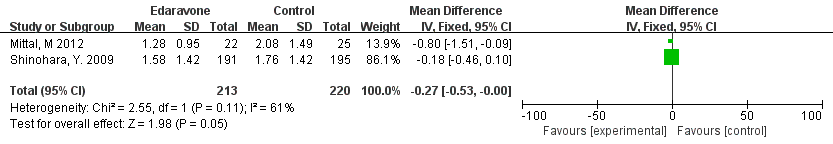

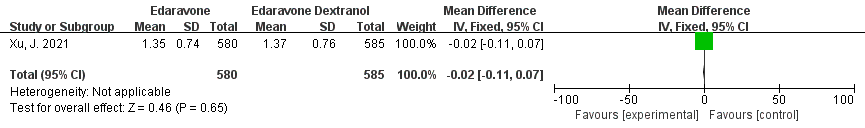

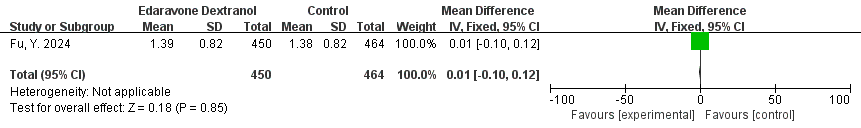

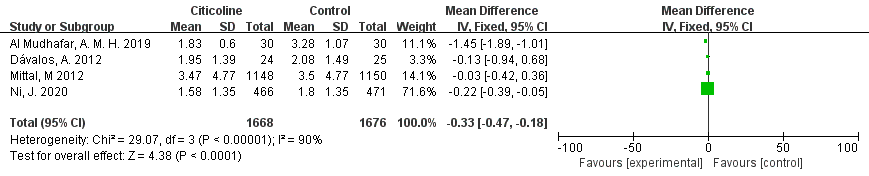

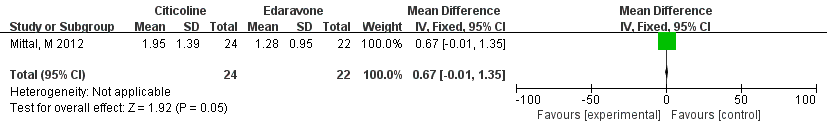

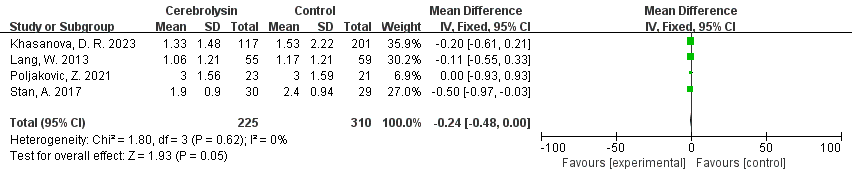
**

**Supplementary Figure 1.** Forest plots of the pairwise meta-analysis for 90-day mRS. mRS, modified Rankin Scale


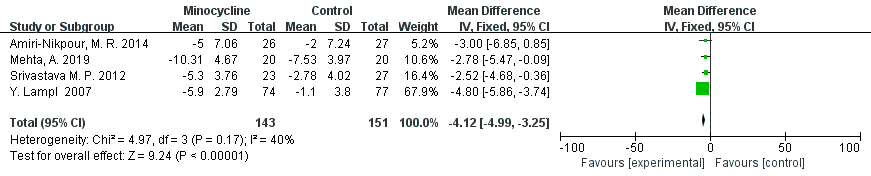

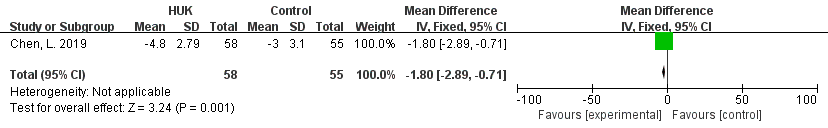

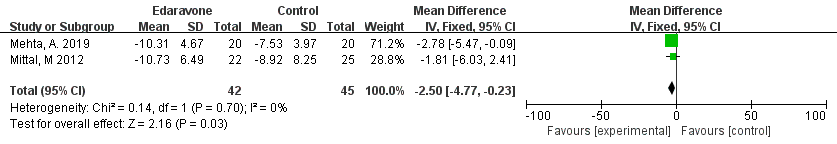

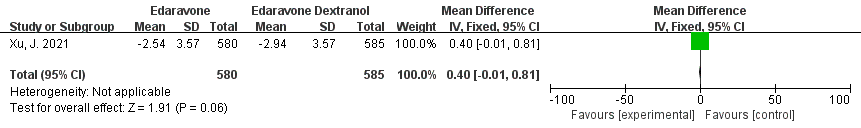

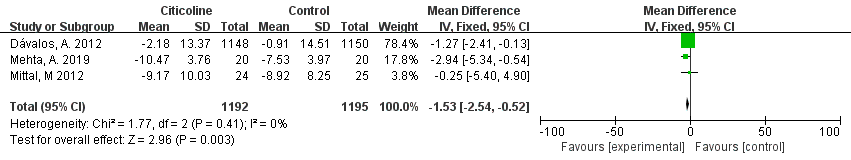

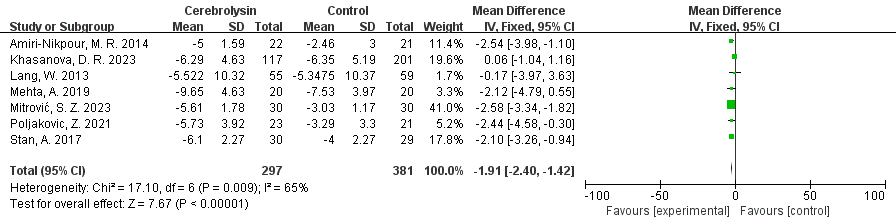

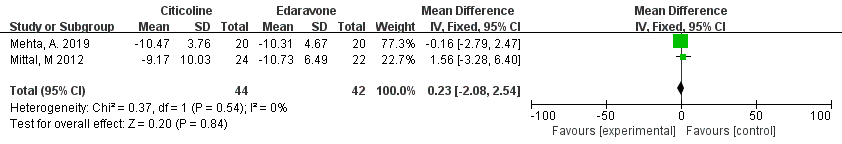

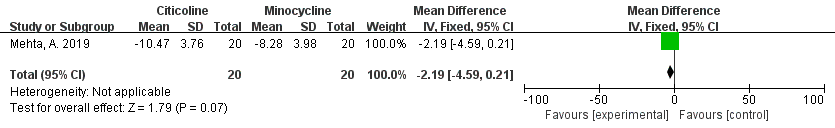

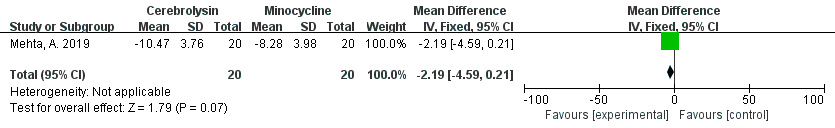

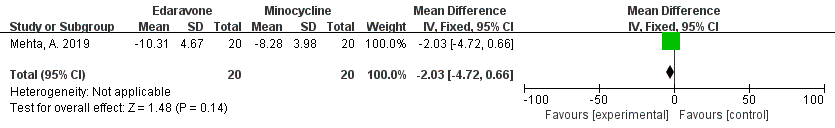

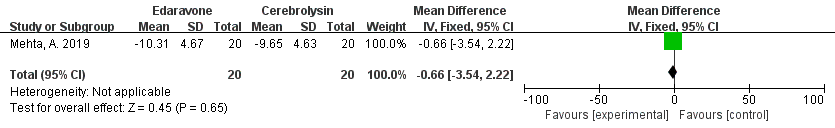


**Supplementary Figure 2.** Forest plots of the pairwise meta-analysis for 90-day NIHSS. NIHSS, National Institutes of Health Stroke Scale.

**
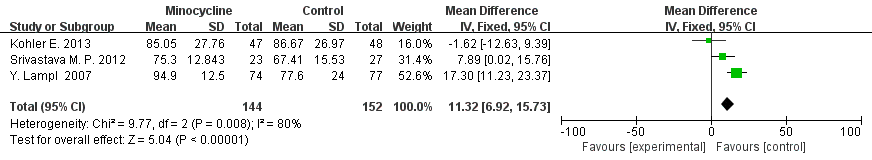

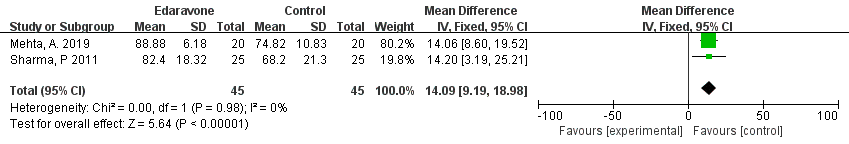

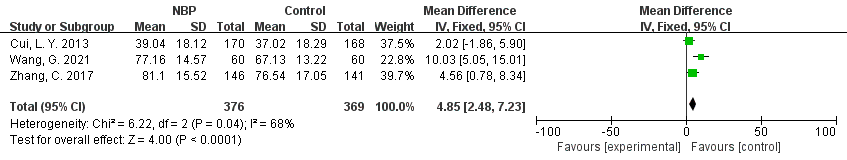

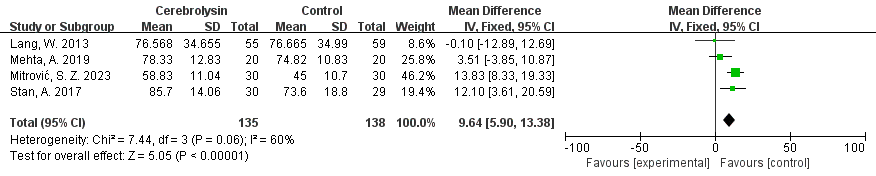

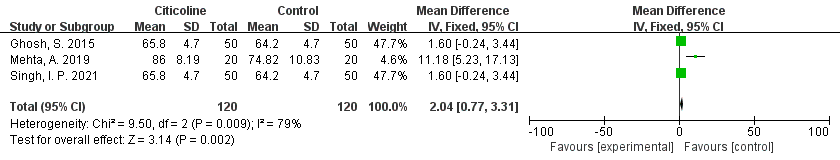

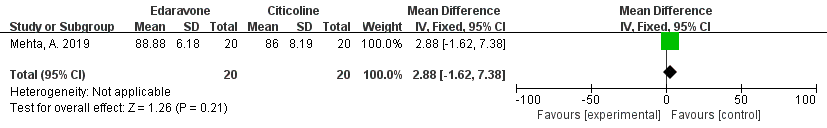

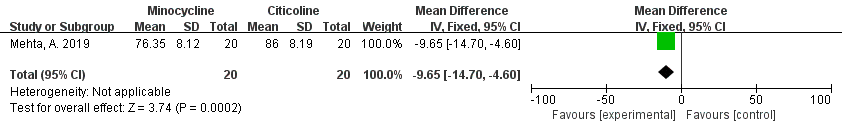

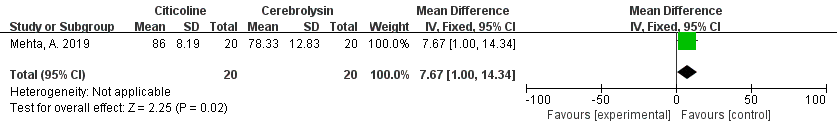

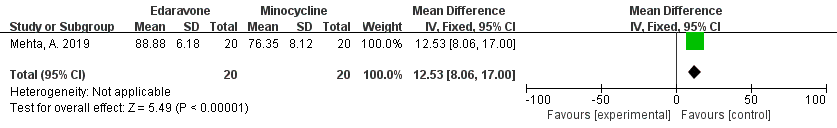

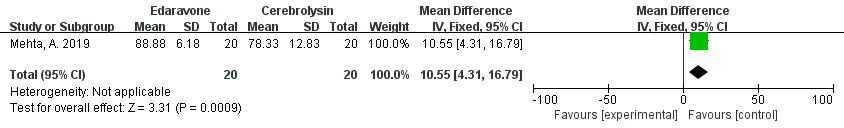

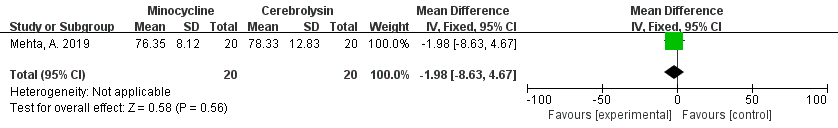
**

**Supplementary Figure 3.** Forest plots of the pairwise meta-analysis for 90-day BI. BI, Barthel Index

**
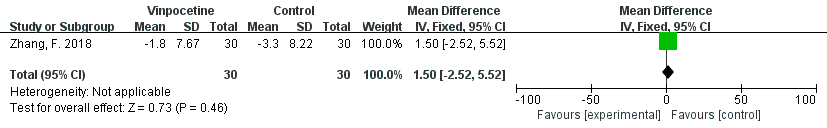

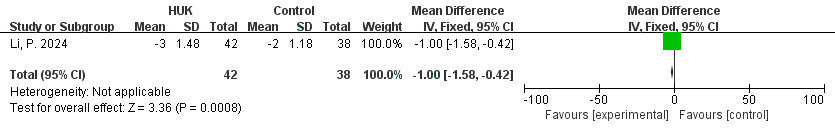

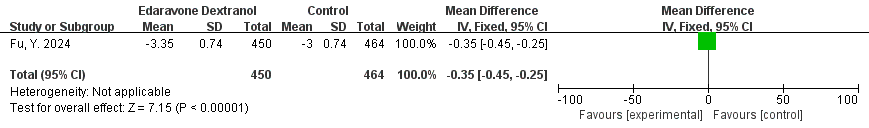

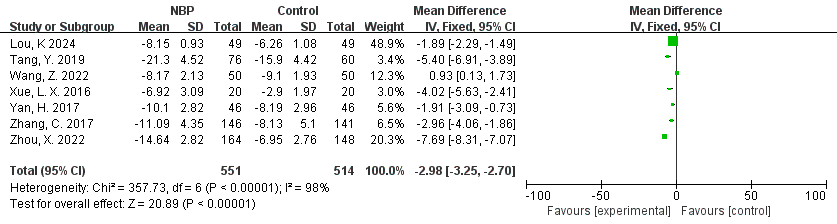

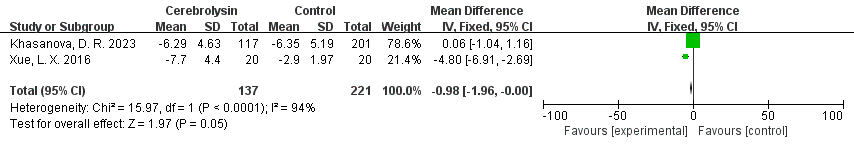

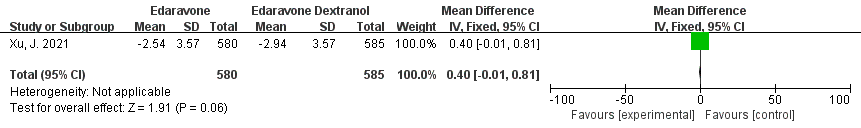

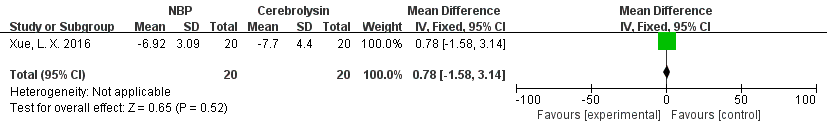
**

**Supplementary Figure 4.** Forest plots of the pairwise meta-analysis for 14-day NIHSS. NIHSS,National Institutes of Health Stroke Scale.


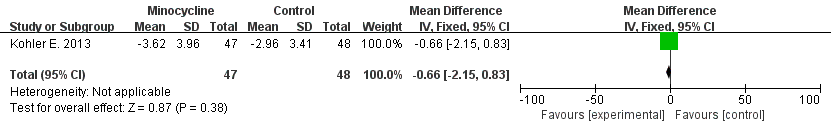

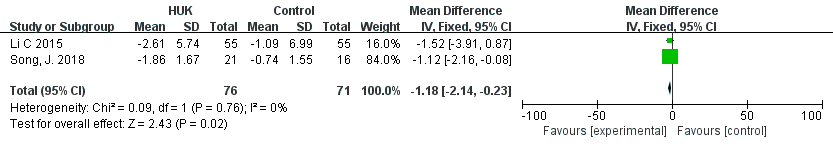

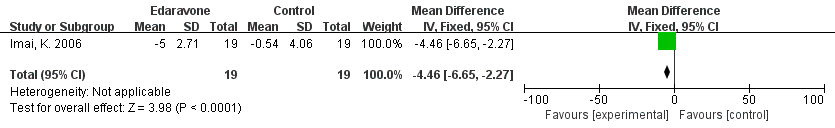

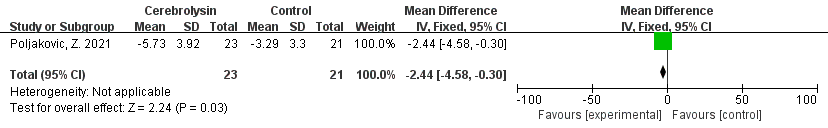


**Supplementary Figure 5.** Forest plots of the pairwise meta-analysis for 7-day NIHSS. NIHSS, National Institutes of Health Stroke Scale.


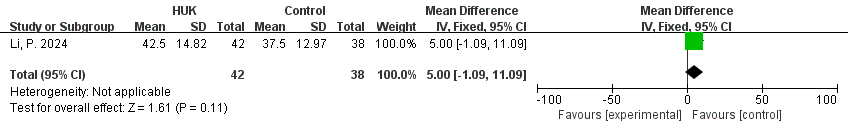

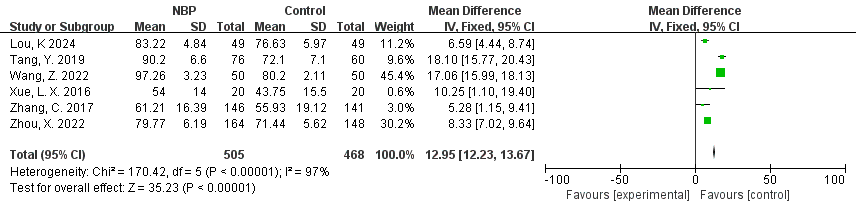

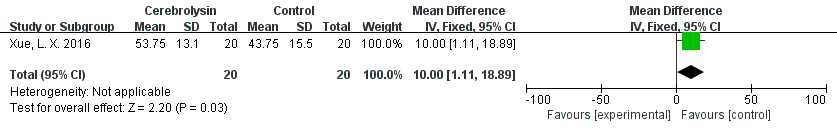

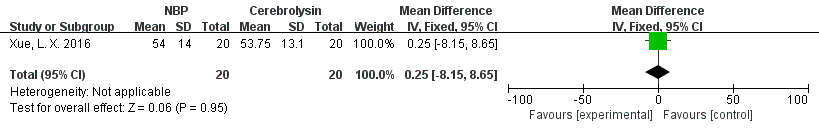


**Supplementary Figure 6.** Forest plots of the pairwise meta-analysis for 14-day BI. BI, Barthel Index

| A 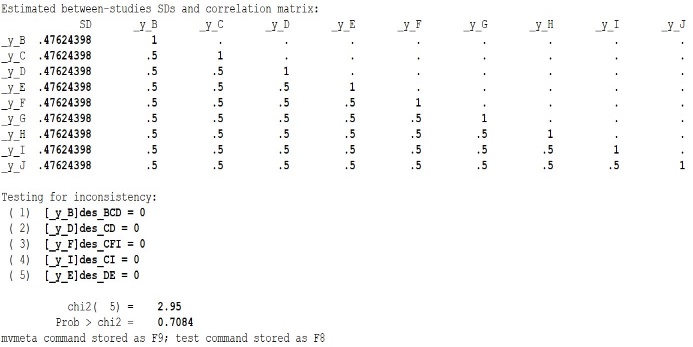 | B 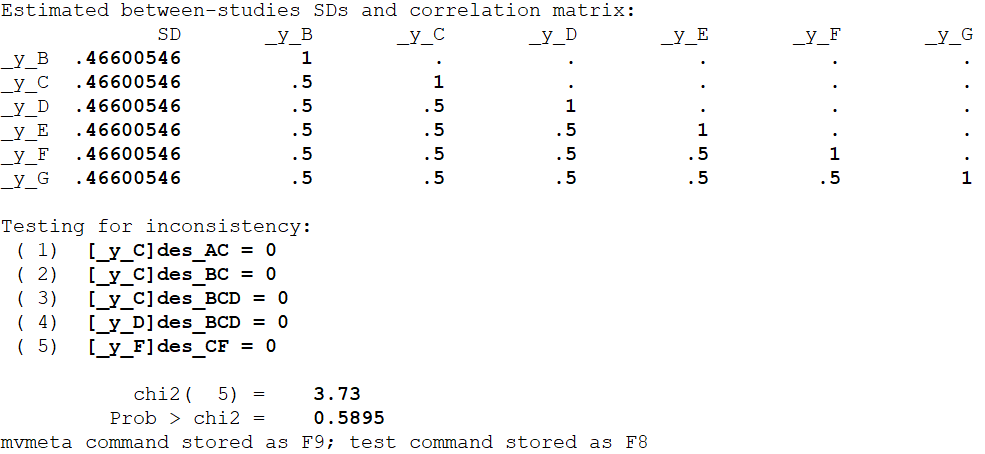 |
| --- | --- |
| C 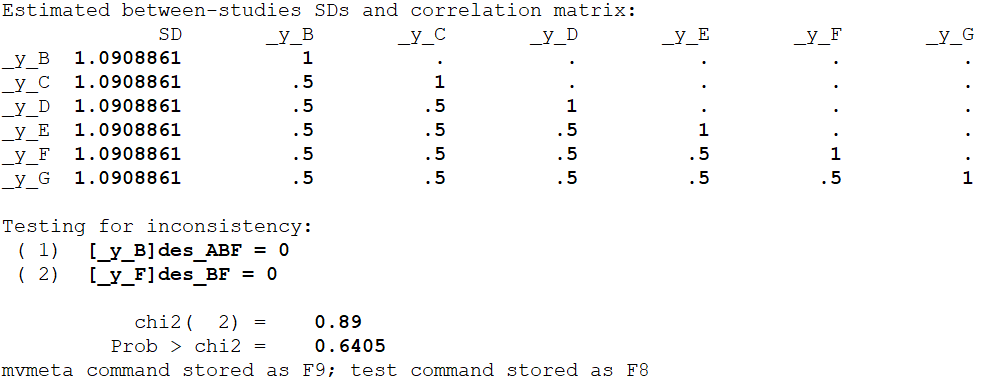 | D 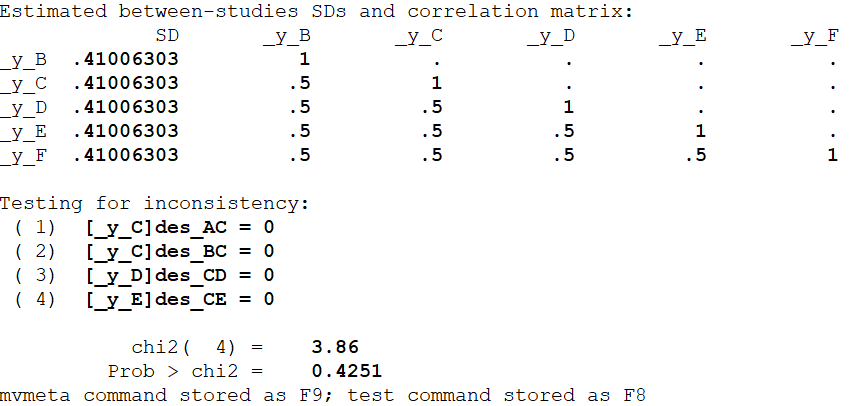 |
| E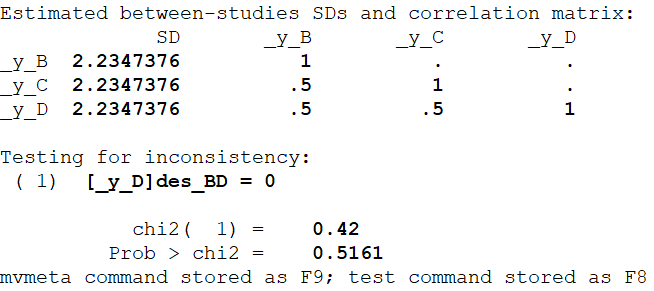 |  |

**Supplementary Figure 7.** (A) 90D-mRS; (B) 90d-NIHSS; (C) 14d-NIHSS; (D) 7d-NIHSS; (D) 90d-BI; (E) 14d-BI.


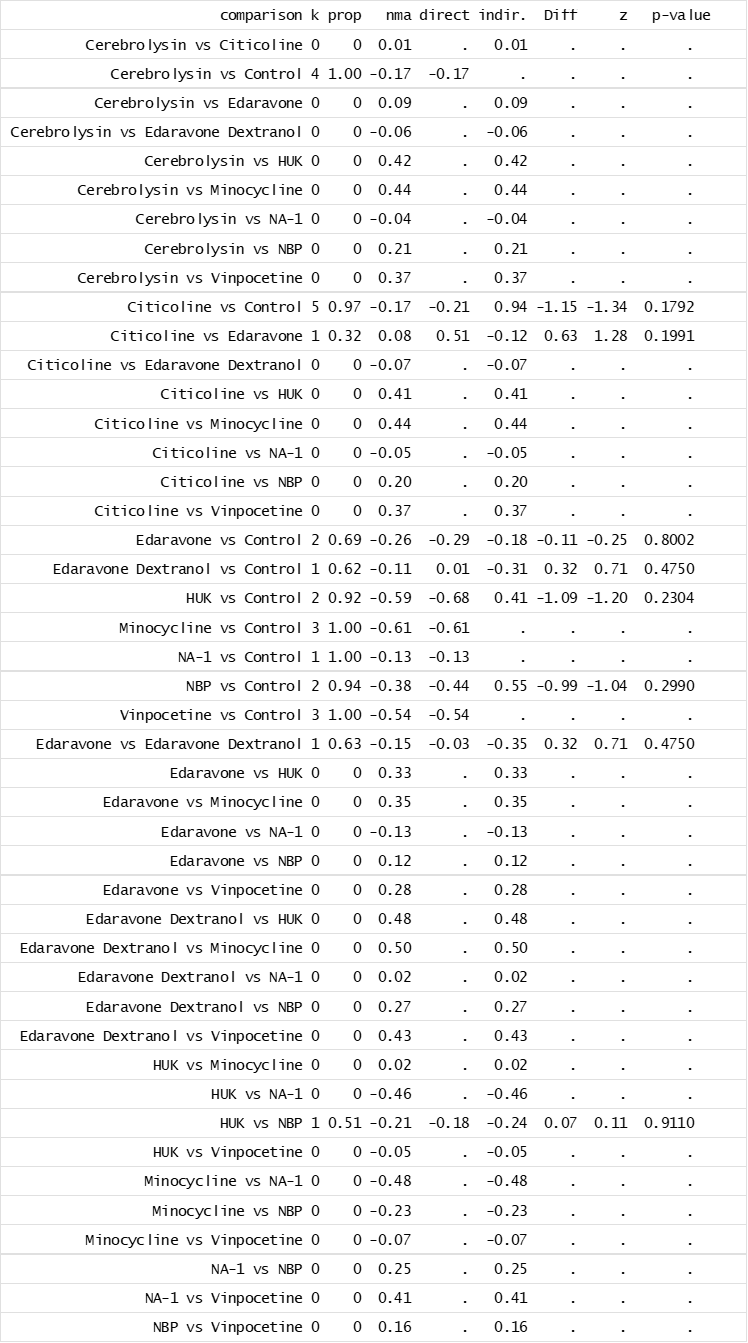


**Supplementary Figure 8.** Local inconsistency test of 90-day mRS

**Supplementary Figure 9.** Local inconsistency test of 90-day NIHSS

**Supplementary Figure 10.** Local inconsistency test of 14-day NIHSS

**Supplementary Figure 11.** Local inconsistency test of 90-day BI

**Supplementary Figure 12.** Local inconsistency test of 14-day BI

**2. Supplementary Tables**

**Supplementary Table 1.** Search strategy (using PubMed as an example).

| Search | Query |
| --- | --- |
| #1 | "Nerinetide"[Title/Abstract] OR "Human urinary kallidinogenase"[ Title/Abstract] OR ("Edaravone"[ Mesh] OR "Norantipyrine" [Title/Abstract] OR "Norphenazone" [Title/Abstract]) OR ("Vinpocetine"[Mesh] OR "Cavinton"[Title/Abstract] OR "Ethyl apovincaminate"[Title/Abstract]) OR ("3-n-butylphthalide"[Mesh] OR "Butylphthalide"[Title/Abstract] OR "n-Butylphthalide"[Title/Abstract] OR "l-NBP cpd"[Title/Abstract]) OR ("Minocycline"[ Mesh] OR "Minocin"[Title/Abstract] OR ("Minox"[Title/Abstrac] OR "Arestin"[Title/Abstract] OR "Dynacin"[Title/Abstract] OR "Klinomycin"[Title/Abstract] OR "Mynocine"[Title/Abstract]) OR ("Cytidine Diphosphate Choline"[ Mesh] OR "Citicoline"[Title/Abstract] OR "CDP-Choline"[Title/Abstract] OR " Citicholine"[Title/Abstract] OR ""[Title/Abstract] OR "Cyticholine "[Title/Abstract])) |
| #2 | ((“Stroke”[Mesh]) OR (((((((((((((((((((((((((((((((((Strokes[Title/Abstract]) OR (Cerebrovascular Accident[Title/Abstract])) OR (Cerebrovascular Accidents[Title/Abstract])) OR (CVA (Cerebrovascular Accident[Title/Abstract]))) OR (CVAs (Cerebrovascular Accident[Title/Abstract]))) OR (Cerebrovascular Apoplexy[Title/Abstract])) OR (Apoplexy, Cerebrovascular[Title/Abstract])) OR (Vascular Accident, Brain[Title/Abstract])) OR (Brain Vascular Accident[Title/Abstract])) OR (Brain Vascular Accidents[Title/Abstract])) OR (Vascular Accidents, Brain[Title/Abstract])) OR (Cerebrovascular Stroke[Title/Abstract])) OR (Cerebrovascular Strokes[Title/Abstract])) OR (Stroke, Cerebrovascular[Title/Abstract])) OR (Strokes, Cerebrovascular[Title/Abstract])) OR (Apoplexy[Title/Abstract])) OR (Cerebral Stroke[Title/Abstract])) OR (Cerebral Strokes[Title/Abstract])) OR (Stroke, Cerebral[Title/Abstract])) OR (Strokes, Cerebral[Title/Abstract])) OR (Stroke, Acute[Title/Abstract])) OR (Acute Stroke[Title/Abstract])) OR (Acute Strokes[Title/Abstract])) OR (Strokes, Acute[Title/Abstract])) OR (Cerebrovascular Accident, Acute[Title/Abstract])) OR (Acute Cerebrovascular Accident[Title/Abstract])) OR (Acute Cerebrovascular Accidents[Title/Abstract])) OR (Cerebrovascular Accidents, Acute[Title/Abstract])) OR (apoplexy[Title/Abstract])) OR (hemiplegia[Title/Abstract])) OR (cerebrovascular disease[Title/Abstract])) OR (cerebral infarction[Title/Abstract])) OR (cerebral hemorrhage[Title/Abstract]))) |
| #3 | #1 AND #2 |

**Table 3 Modified Rankin Scale (mRS)**

| **Score** | **Description** |
| --- | --- |
| **0** | No symptoms. |
| **1** | No significant disability; able to carry out all usual activities despite symptoms. |
| **2** | Slight disability; unable to carry out all previous activities but able to look after own affairs without assistance. |
| **3** | Moderate disability; requiring some help, but able to walk without assistance. |
| **4** | Moderately severe disability; unable to walk without assistance and unable to attend to own bodily needs without assistance. |
| **5** | Severe disability; bedridden, incontinent, and requiring constant nursing care and attention. |
| **6** | Death. |

**Table 4 National Institutes of Health Stroke Scale (NIHSS)**

| **Category** | **Description** | **Score Range** |
| --- | --- | --- |
| **1a. LOC: Alertness** | 0 = Alert, 1 = Not alert, 2 = Requires repeated stimulation, 3 = Coma | 0–3 |
| **1b. LOC Questions** | 0 = Answers both correctly, 1 = One correct, 2 = None correct | 0–2 |
| **1c. LOC Commands** | 0 = Obeys both, 1 = One correct, 2 = None correct | 0–2 |
| **2. Best Gaze** | 0 = Normal, 1 = Partial palsy, 2 = Forced deviation | 0–2 |
| **3. Visual Fields** | 0 = No loss, 1 = Partial hemianopia, 2 = Complete hemianopia, 3 = Bilateral blindness | 0–3 |
| **4. Facial Palsy** | 0 = Normal, 1 = Minor paralysis, 2 = Partial paralysis, 3 = Complete paralysis | 0–3 |
| **5. Motor Arm** | 0 = No drift, 1 = Drift, 2 = Effort against gravity, 3 = No effort, 4 = No movement | 0–4 each side |
| **6. Motor Leg** | Same as Motor Arm criteria | 0–4 each side |
| **7. Limb Ataxia** | 0 = None, 1 = One limb, 2 = Two limbs | 0–2 |
| **8. Sensory** | 0 = Normal, 1 = Mild loss, 2 = Severe loss | 0–2 |
| **9. Best Language** | 0 = Normal, 1 = Mild aphasia, 2 = Severe aphasia, 3 = Mute/global aphasia | 0–3 |
| **10. Dysarthria** | 0 = Normal, 1 = Mild, 2 = Severe | 0–2 |
| **11. Extinction/Inattention** | 0 = Normal, 1 = Neglect in one modality, 2 = Severe neglect | 0–2 |

**Table 5 Barthel Index(BI)**

| **Activity** | **Scoring Criteria** | **Score** |
| --- | --- | --- |
| **Feeding** | 0 = Unable, 5 = Needs help, 10 = Independent | 0–10 |
| **Bathing** | 0 = Dependent, 5 = Independent | 0–5 |
| **Grooming** | 0 = Needs help, 5 = Independent | 0–5 |
| **Dressing** | 0 = Dependent, 5 = Needs help, 10 = Independent | 0–10 |
| **Bowel Control** | 0 = Incontinent, 5 = Occasional accident, 10 = Continent | 0–10 |
| **Bladder Control** | Same as Bowel Control criteria | 0–10 |
| **Toilet Use** | 0 = Dependent, 5 = Needs help, 10 = Independent | 0–10 |
| **Transfers (Bed to Chair)** | 0 = Unable, 5 = Major help, 10 = Minor help, 15 = Independent | 0–15 |
| **Mobility** | 0 = Immobile, 5 = Wheelchair, 10 = Walks with help, 15 = Independent | 0–15 |
| **Stairs** | 0 = Unable, 5 = Needs help, 10 = Independent | 0–10 |
